# Neoantigen immune responses in healthy volunteers: insights from multiple integrated keyhole limpet hemocyanin challenge studies on repeated immunization and response covariates

**DOI:** 10.3389/fphar.2025.1717333

**Published:** 2026-01-02

**Authors:** Micha N. Ronner, Marieke L. De Kam, Silke Van Reeuwijk, Jacobus Burggraaf, Manon A. A. Jansen, Matthijs Moerland

**Affiliations:** 1 Centre for Human Drug Research, Leiden, Netherlands; 2 Leiden University Medical Center, Leiden, Netherlands

**Keywords:** KLH, keyhole limpet hemocyanin, adaptive immune system, challenge model, standardized analysis, repeat immunizations, recall response, local skin response

## Abstract

**Introduction:**

Investigational immunomodulatory drugs are often initially investigated in healthy volunteers, which lack drug-target engagement biomarkers, hampering evaluation of pharmacodynamic effects. A model such as the Keyhole Limpet Hemocyanin (KLH) neoantigen challenge induces a well-controlled adaptive immune response and can be used to study pharmacodynamic effects in healthy volunteers. To increase our understanding of sources of variability in KLH-induced responses, we integrated KLH data from multiple studies executed at our research institute and investigated the contribution of covariates to KLH challenge responses.

**Methods:**

We performed a pooled analysis of seven KLH trials with standardized methodology and design. Included trials immunized participants with KLH one, two or three times, with an interval of 2 weeks, followed by subsequent intradermal KLH administration 2–3 weeks after the final immunization. Differences and correlations were calculated between anti-KLH IgM and IgG and skin perfusion, flare, and erythema. A mixed effects model analysis was performed to estimate variances and determine the effect of the number of immunizations and the systemic and local biomarkers on the sample size. Finally, the effect of covariates age, sex, and body mass index was analyzed.

**Results:**

A total of 68 participants were included: 42 participants were immunized with KLH once, 5 participants twice, and 21 participants three times. Three times immunization resulted in significantly higher antibody levels, cutaneous perfusion, and flare. A study design incorporating three immunizations and skin imaging at 24 h was found to have the most robust statistical power. Lastly, the systemic KLH response correlated negatively with age and positively with local KLH responses.

**Conclusion:**

This analysis provides a basis for the design of future clinical studies with KLH challenges. These data provide further insights into the performance of the human KLH challenge, which will facilitate the implementation of this immune challenge model in future clinical pharmacology studies.

## Highlights

- The effect size of both the systemic antibody response and the perfusion-directed local recall response are dose-dependent, with effect size increasing when increasing the number of prior KLH immunizations from one to three.
- Increasing the number of KLH immunizations, from one to three, increased the power to detect a statistically significant effect in the planimetric skin response, but decreased the power to detect an effect on the humoral response.
- A study design incorporating multiple immunizations and skin imaging at 24 h results in the most robust statistical power to detect a significant effect between groups.
- Systemic and local outcome parameters of the KLH challenge are significantly positively correlated.

## Introduction

Many investigational drugs fail clinical development in phase 2 due to a lack of effectivity (Wong et al., 2019). The rate of failure in phase 2 can be significantly reduced by investigating pharmacodynamic effects of novel compounds early in their clinical development. Classically, early phase clinical trials primarily investigate the tolerability and pharmacokinetics of a novel compound and are often executed in healthy volunteers. In the field of immunology, these volunteers often lack expression of drug targets or activity of relevant pathophysiological pathways, hampering evaluation of pharmacodynamic effects of immunomodulatory compounds. This limitation can be addressed by inducing well-controlled immune responses, either *in vitro* or *in vivo*, through immune challenges. These challenges induce an innocuous, transient, and controlled immune response and are increasingly used in both preclinical and early clinical development studies to test efficacy of novel immunomodulatory compounds (Swaminathan et al., 2014; Buters et al., 2022; Saghari et al., 2022; van den Noort et al., 2025). These compounds are tested by administering an auxiliary substance to induce an innate or adaptive immune response. One auxiliary substance used for adaptive immune challenges is the neoantigen Keyhole Limpet Hemocyanin (KLH). The KLH challenge model entails that participants are first immunized with KLH, after which the systemic response can be monitored in blood using cellular biomarkers and molecular biomarkers such as IgM and IgG titers. Subsequently, KLH is administered intradermally to elicit a local recall response, which can be assessed at the cellular and molecular levels through skin tissue analysis, as well as non-invasively through skin imaging. Because there is not one sole effective dosing regimen for the immunization and local administration, and not a standardized way to monitor and quantify the KLH response, the KLH challenge model suffers from variability between studies and research groups (Saghari et al., 2022). This variability hampers the application of the model for clinical evaluation of novel immunomodulatory compounds. To increase our understanding of sources of variability in KLH-induced responses, we integrated KLH data from multiple studies executed at our research institute and investigated the contribution of covariates to KLH challenge responses.

When using the KLH challenge model, the ideal formulation, dose, and regimen elicit minimal adverse events while producing a significant desired response. At our institution we selected formulation, adjuvant, dose, and regimen, based on KLH data available in the public domain (Miller et al., 2005; Milgrom et al., 2012; Swaminathan et al., 2014; Saghari et al., 2022). Specifically, subunit KLH combined with aluminum as adjuvant was chosen because of its safety, tolerability, and ability to elicit detectable systemic and local immunological responses (Spazierer et al., 2009; Swaminathan et al., 2014; Saghari et al., 2022). To maximize consistency, methodology and study design were standardized across studies. Furthermore, repeat immunization has been reported to result in stronger systemic responses than single immunization, even when the total dosage of administered KLH is the same (Saghari et al., 2022). Commonly, repeated immunizations are given with intervals of 1–3 weeks, and anti-KLH IgM and IgG titers start to increase 7–14 days after immunizations (Saghari et al., 2022). Therefore, we incorporated a 14-day window between immunizations in KLH challenge studies with repeated immunization.

While the effect of repeated immunizations on the systemic KLH response has previously been reported, the effect on the local response has not been studied in detail (Spazierer et al., 2009; Boulton et al., 2012; Giesecke et al., 2018; Saghari et al., 2022). Therefore, sensitive imaging-based techniques were implemented to objectively quantify the local KLH recall response upon a dermal challenge. The combination of resulting functional endpoints, such as skin perfusion and erythema, with a standardized approach to elicit KLH responses enabled robust analysis of correlations across the diverse KLH-driven biomarkers, both systemic and tissue-specific.

The primary aim of this pooled analysis was to evaluate the relationship between the number of KLH immunizations and the local KLH response size. Additionally, we sought to confirm previous findings indicating repeated KLH immunizations to result in a stronger systemic response. We studied the correlation between the systemic and local KLH response, and evaluated the impact of age, sex, and body mass index (BMI) on the KLH response. Altogether, we present the lessons learned from integrating KLH response data from multiple studies with a standardized methodology, with the aim to further improve the KLH challenge model for future application in clinical studies evaluating novel immune-targeted compounds.

## Materials and methods

### Participants

This analysis included participants from seven separate clinical trials labeled as study A to G. Participants were considered eligible for inclusion if they participated in a healthy volunteer KLH trial at the Centre for Human Drug Research (CHDR; Leiden, the Netherlands). Standard inclusion criteria for KLH studies at CHDR were: Healthy volunteer, as verified by extensive medical screening; Fitzpatrick skin-type I-III. Standard exclusion criteria were: prior exposure to KLH or the Schistosoma parasite, or a history of keloid or excessive scar formation. Dependent on the study type, the inclusion criteria related to age and sex differed. Studies A, B, E and F only included male participants. For studies A, B, E and G participants aged 18–45 years were included. In studies C and D, participants aged 18–60 years were included. In study F, participants aged 18–55 years were included (Supplementary Table S1). All included studies were approved by a medical ethical committee and were registered in the European Union Drug Regulating Authorities Clinical Trials Database (A: EUCTR2016-004839-20-NL; B: EUCTR2017-000084-32-NL; C: EUCTR2019-000166-38-NL; D: EUCTR2018-002658-65-NL; E: EUCTR2021-004136-28-NL; F: EUCTR2021-003021-30-NL; G: EUCTR2022-000975-37-NL). All participants provided written informed consent prior to study participation.

### Study design

**FIGURE 1:**
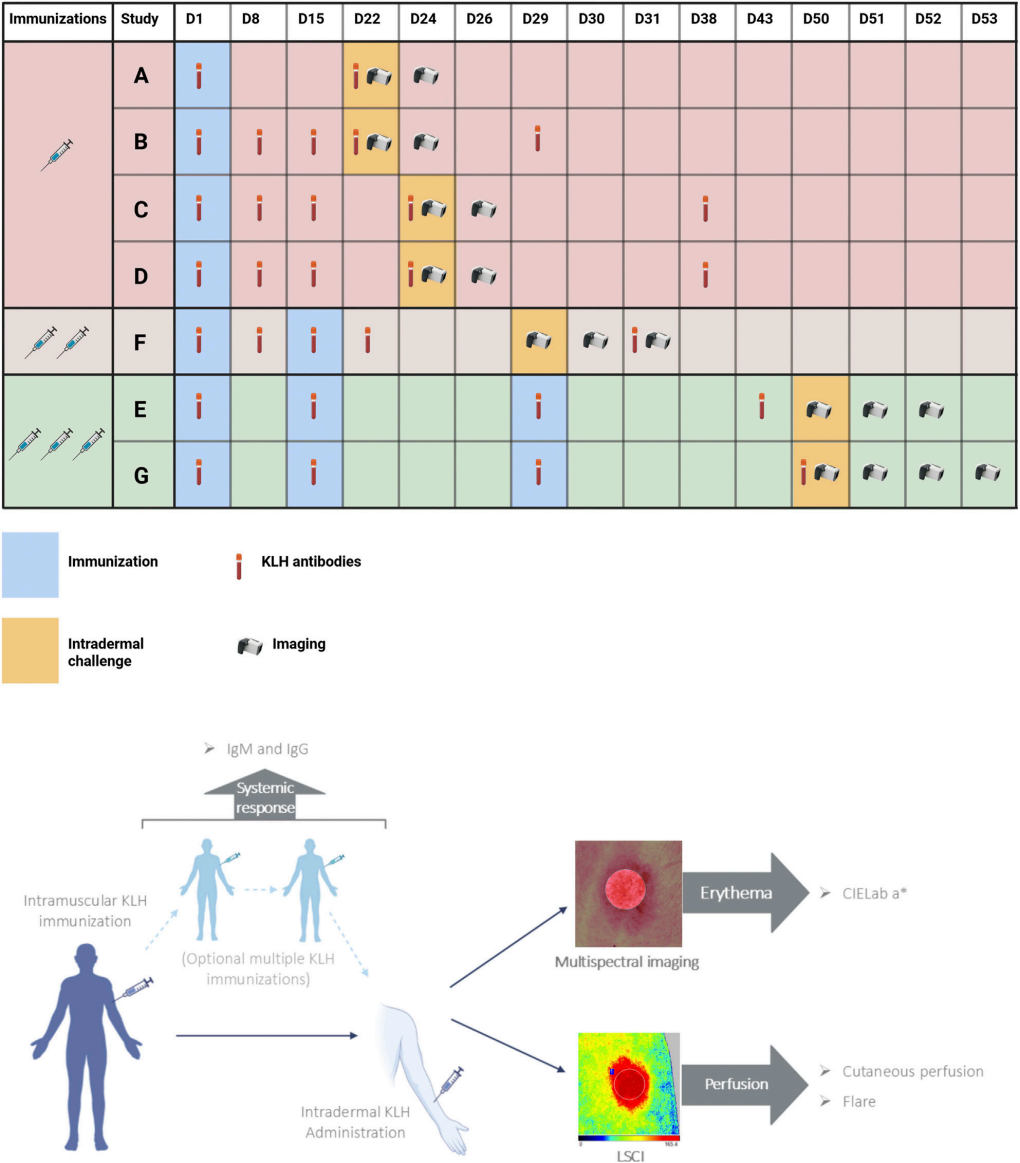
Study design. Schedule of assessments of seven studies depicted in a timeline, where participants of studies A, B, C and D were immunized once (light-red), participants of study F were immunized twice (light-brown) and participants of studies E and G were immunized three times prior to intradermal KLH administration. KLH antibody measurements included anti-KLH IgM and anti-KLH IgG. Imaging included laser speckle contrast imaging (PeriCam PSI System, Perimed AB, Järfälla, Sweden) and multispectral imaging (Antera 3D; Miravex, Dublin, Ireland). Abbreviations: KLH, keyhole limpet hemocyanin; LSCI, laser speckle contrast imaging. Created with BioRender.com.

This analysis included participants from seven randomized controlled clinical trials. Only participants receiving KLH and no other investigational treatment were included in this pooled analysis. A schematic overview of the different trials can be found in Figure 1. In all studies, participants were immunized with KLH, which was administered intramuscularly in the deltoid muscle of the upper arm in a dosage of 100 µg subunit KLH (Immucothel, Biosyn Corporation, Carlsbad CA, United States) adsorbed to 1,320 µg aluminum hydroxide (Alhydrogel, Brenntag AG, Essen, Germany). Participants were immunized one, two, or three times with an interval of 2 weeks. Subsequently, 1 µg KLH was intradermally administered in the volar side of the lower forearm between 14 and 23 days after the final immunization (Figure 1). All but one of the trials included an additional intradermal saline injection as a control area for imaging endpoints. Trial B included an untreated control area instead.

### Outcome parameters

#### Systemic immune response

All trials measured anti-KLH IgM and IgG antibodies, and antibody data was available for this analysis of six trials, because antibody analysis of one trial (F) was measured at a laboratory with different methodology. Antibodies were measured repeatedly, starting at baseline and at different subsequent timepoints based on the trial (Figure 1). Trial A measured antibodies 21 days after immunization. Trial B measured antibodies 7, 14, 21 and 28 days after immunization. Trials C and D measured antibodies 7, 14, 23 and 37 days after immunization. Trial E measured antibodies at 14, 28 and 42 days after initial immunization. Trial G measured antibodies 14, 28 and 49 days after initial immunization. In the trials that included KLH immunizations scheduled on the same day as antibody measurements, blood was drawn prior to immunization with KLH. Enzyme-linked immunosorbent assay (Ardena Bioanalytical Laboratory, Assen, the Netherlands) was used to quantify anti-KLH IgM and IgG antibodies. Antibody values were reported as a ratio compared to the baseline measurement, which was set at 100.

#### Local immune response

##### Cutaneous perfusion

Cutaneous perfusion was quantified using laser speckle contrast imaging (LSCI; PeriCam PSI System, Perimed AB, Järfälla, Sweden), as previously described (Saghari et al., 2021). Briefly, participants first acclimatized to room temperature for 15 min, after which recordings of a target area of 7 by 7 cm on the volar side of the lower forearm were captured using dedicated software (Pimsoft, Perimed AB). This software was used to capture a recording of at least 30 s with a frame rate of 21 images per second. Subsequently, recordings were analyzed by means of a region of interest (ROI) placed on the target area. All recordings were analyzed using a ROI of the same shape and size to ensure comparability between results of the different trials. Mean cutaneous perfusion within the ROI was quantitatively assessed and expressed in perfusion units (PUs). Homogeneity of the reaction was expressed as flare, defined as the area within the ROI that is 1 standard deviation above or below the mean cutaneous perfusion. The flare was quantitatively assessed and also expressed in PUs.

##### Erythema

Erythema was measured by means of multispectral imaging using the Antera 3D camera (Antera 3D; Miravex, Dublin, Ireland), as previously described (Saghari et al., 2021). Images of 5 by 5 cm, without exposure to ambient light, were made of the volar side of the lower forearm intradermally injected with KLH. Images were analyzed using dedicated software to create a ROI which was applied to the target area. All images were analyzed using a ROI of the same shape and size to ensure comparability between results of the different trials. Erythema was quantitatively assessed and expressed as CIELab a* in arbitrary units (AUs) (Ly et al., 2020).

Initially, cutaneous perfusion and erythema were assessed only at baseline and 48 h after intradermal KLH administration (Trials A, B, C, D, Figure 1). When trial designs included multiple KLH immunizations, additional timepoints were included to measure the local skin response. Trial E included measurements at 0.5, 2, 6, 24 and 48 h post intradermal KLH administration. Trial F included measurements at 0.5, 24 and 48 h. Trial G included measurements at 4, 24, 48 and 72 h.

### Statistical analyses

#### Pooling the data

Since this analysis incorporated data collected from multiple trials executed between 2017 and 2023, the data first had to be pooled into a single database prior to analysis. Data was combined into a single database using R statistical software (version 4.3.2) (R Core Team, 2023). When combining the data, the anti-KLH IgM and IgG values of trial B required a 100-fold transformation, as the baseline measurement value was originally set at 1 instead of 100. Antibody levels were reported as the optical density ratio of a sample compared to baseline. Due to KLH being a neoantigen, it is the assumption that no KLH antibodies are present during the baseline measurements. Therefore, the optical density of the baseline measurement was heavily dependent on the optical density of the buffer solution that was used. Because of the substantial impact of the buffer on the baseline measurement and because subsequent measurements were originally expressed as a ratio over baseline, the buffer had a substantial influence on the reported ratios. As the optical density of the buffer differed per study but not within study, the measurements were corrected per study based on the optical density of the buffer. This allowed for comparison of the antibody levels between studies. All imaging data was reanalyzed with the same ROI prior to pooling the data, to ensure consistency of analysis.

#### Analysis of the systemic and local immune response

Baseline characteristics were calculated per immunization category, namely, one, two, or three times immunized with KLH (Table 1). For statistical testing of differences in antibody levels between the one-, two-, and three-times KLH-immunized participants, the antibody measurement closest to 3 weeks after the last KLH immunization was used. Statistical testing of cutaneous perfusion, flare and erythema between the different immunization categories (once, twice or three times) was done by comparing the outcomes at 48 h post-intradermal KLH with a one-way analysis of variance with baseline and control area response as covariate (ANCOVA). This was repeated with outcomes at 24 h, if data from 24 h was available. All statistical programming was conducted by a trained statistician with SAS 9.4 software for Windows (SAS Institute Inc., Cary, NC, United States). For all analyses diagnostic plots and normality tests (Shapiro-Wilck) were performed to check if the assumptions of the analysis method were met. Antibody levels were log-transformed before analysis, therefore estimated differences are presented in percentages.

**TABLE 1:** Baseline characteristics.

Characteristic	All participants	One immunization	Two immunizations	Three immunizations
Age (years)				
N	68	42	5	21
Mean (SD)	29.3 (10.8)	30.9 (12.3)	31.2 (12.6)	25.6 (5.27)
Median	25	25	25	26
Min	18	18	20	18
Max	59	59	51	39
Height (cm)				
N	68	42	5	21
Mean (SD)	180 (8.5)	179 (8.66)	181 (8.93)	183 (7.59)
Median	182	180	176	183
Min	162	162	174	167
Max	196	195	195	196
Weight (kg)				
N	68	42	5	21
Mean (SD)	77.6 (11.8)	77.7 (12)	77.6 (4.24)	77.4 (13)
Median	76.5	77.4	78.6	73.4
Min	53.2	53.2	71.4	56.6
Max	106	101	81.8	106
BMI (kg/m2)				
N	68	42	5	21
Mean (SD)	23.8 (3.1)	24.3 (3.14)	23.2 (1.92)	23 (3.16)
Median	23.6	24.2	24	22
Min	18.7	18.7	20	19
Max	33.4	33.4	25	29.9
Sex				
Male	0.74 (50/68)	0.62 (26/42)	1.00 (5/5)	0.90 (19/21)

Demographics of study participants, summarized for all participants and separated per number of KLH immunizations. Abbreviations: BMI, body mass index; CM, centimeters; KG, kilogram; KLH, keyhole limpet hemocyanin; SD, standard deviation.

#### Estimates of variance analysis

A repeated measures mixed effects model analysis was performed to estimate variances within the one- and three-times immunized groups and to determine the effect of the number of immunizations and the systemic and local biomarkers on the sample size required to detect a significant effect of a compound counteracting the KLH response. For this purpose, the inter-participant standard deviation was estimated with a model with pre-value and, if applicable control area response, as covariances, a repeated factor subject (the subject of the repeated measures being number of immunizations group) within the number of immunizations group and a variance components variance/covariance structure. Separate inter-participant standard deviations were calculated for the antibody levels of the one- and three-times immunized participants as well as for the 24 h and 48 h measurements. For the antibody levels, the antibody measurement closest to 3 weeks after the last KLH immunization was used. Sample sizes were calculated based on a parallel study design with a two-sided significance of 0.05 and a power of 0.80.

#### Correlation analyses: systemic and local outcome parameters

The correlation between primary outcome parameters was tested using Pearson correlations, in case of antibody levels on log-transformed values. Correlations, with p-values, between anti-KLH IgM and IgG, cutaneous perfusion and flare, cutaneous perfusion and erythema, flare and erythema, antibodies and cutaneous perfusion, antibodies and flare, and antibodies and erythema were calculated. For antibody levels, the antibody measurement closest to 3 weeks after the last KLH immunization was used.

#### Correlation analyses: covariates age, sex, and body mass index

Continuous covariates age and BMI were graphically explored by scatter graphs including the primary outcome measurements. The correlation of the covariate with the primary outcome measurement was tested and calculated using Pearson correlations, in case of antibody level data on log-transformed values. Differences between the sexes on the primary outcome measurements were graphically explored by boxplots. For graphs and correlations with antibody levels, the antibody measurement closest to 3 weeks after the last KLH immunization was used.

## Results

### Baseline characteristics

A total of 68 participants were included in this analysis, of which 42 were immunized once with KLH, 5 participants were immunized twice, and 21 participants were immunized three times. Baseline characteristics were calculated per immunization category and showed a skewing towards male participants (Table 1).

### Systemic response

**FIGURE 2:**
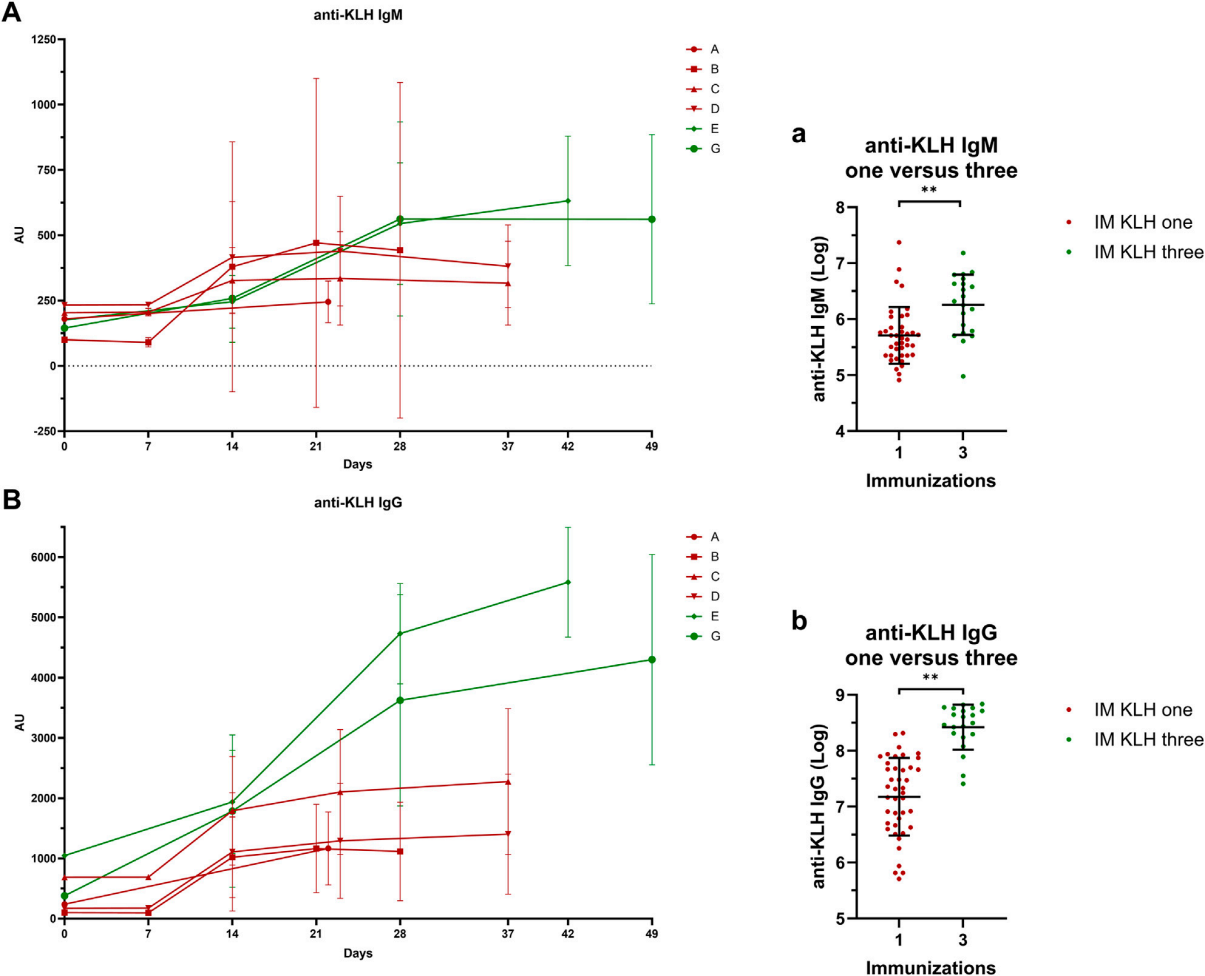
Systemic response: Antibody titers IgM A/a and IgG B/b anti-KLH antibody titers for studies A, B, C, D, E and G. Studies in dark-red represent participants immunized once with KLH, studies in green represent participants immunized three times with KLH. Single-immunized participants were immunized only on Day 1. Three-times immunized participants were immunized with KLH on Day 1, 15 and 29. If antibody titer measurements and KLH immunizations took place on the same day, blood for antibody titer analysis was drawn prior to KLH immunization. In **(A)** and **(B)**, the mean ± standard deviation are shown per study per timepoint. Log-transformed values of the antibody measurement closest to 3 weeks after the last KLH immunization were compared between the one- and three-times KLH-immunized participants a/b. The log-transformed individual measurements are shown and include the mean ± standard deviation a/b. Asterisks indicate statistical significance between the three-times immunized group and the single immunized group. ** indicates a significance of *p* < 0.01. Abbreviations: AU, arbitrary units; IM, intramuscular; KLH, keyhole limpet hemocyanin.

KLH antibody levels increased with multiple immunizations. Both IgM (estimated difference 73%; 95% CI, 31%–128%; *p* = 0.0002) and IgG (estimated difference 247%; 95% CI, 150%–382%; *p* < 0.0001) levels were significantly higher after three immunizations as compared to after one immunization (Figure 2).

### Local response

#### Cutaneous perfusion and erythema

**FIGURE 3:**
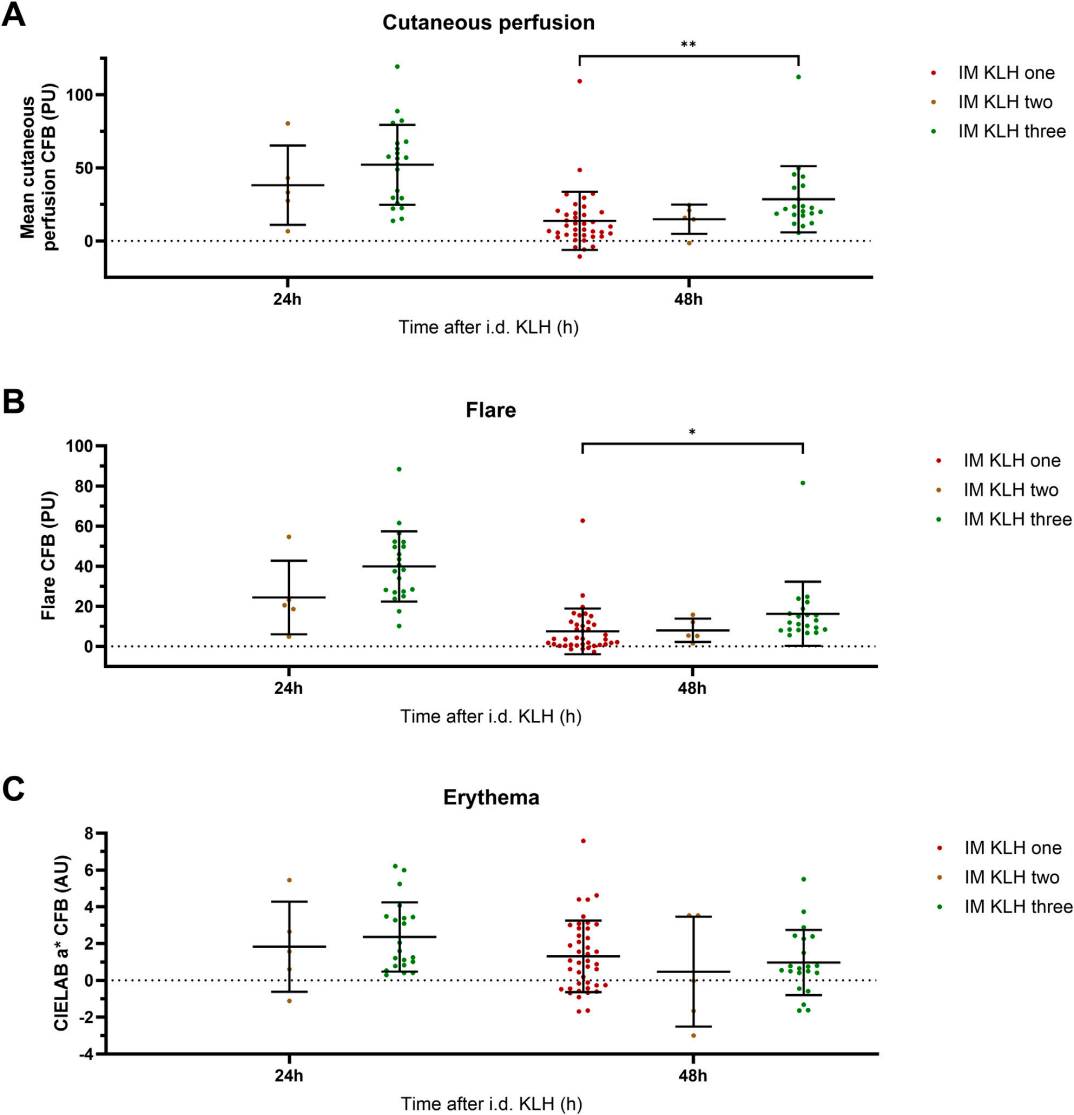
Local response: Perfusion, flare, and erythema. The local response to intradermal KLH as measured by means of laser speckle contrast imaging (LSCI; PeriCam PSI System, Perimed AB, Järfälla, Sweden) and multispectral imaging (Antera 3D; Miravex, Dublin, Ireland) is depicted as cutaneous perfusion (LSCI) **(A)**, flare (LSCI) **(B)**, and erythema (Antera 3D) **(C)**. Single KLH-immunized participants are depicted in dark-red, two-times immunized participants in brown and three-times immunized participants in green. Only the timepoints at which a comparison could be made between different numbers of immunizations are depicted. The individual measurements of the skin response are presented as change from baseline values and include the mean ± standard deviation. The limited sample size of the two-times immunized group resulted in relatively large inter-participant variability. Asterisks indicate statistical significance between the three-times immunized group and the single immunized group. * indicates a significance of *p* < 0.05; ** indicates a significance of *p* < 0.01. Abbreviations: AU, arbitrary units; CFB, change from baseline; h, hours; i.d., intradermal; IM, intramuscular; KLH, keyhole limpet hemocyanin; PU, perfusion units.

The number of KLH immunizations prior to local intradermal KLH administration determined the level of cutaneous perfusion. A significant overall effect of the number of KLH immunizations on cutaneous perfusion at 48 h was observed (*p* = 0.0016) (Figure 3). Additionally, three prior KLH immunizations lead to significantly higher cutaneous perfusion (least square means (LSM) difference 22.1 PU; 95% CI, 10.5 to 33.8; *p* = 0.0004) and flare (LSM difference 11.2 PU; 95% CI, 1.5 to 20.8; *p* = 0.0238) at 48 h than one prior KLH immunization. No other significant effects were detected of the number of KLH immunizations on perfusion, flare, or erythema (Figure 3). In the studies that included multiple timepoints after intradermal KLH administration, a peak in cutaneous perfusion and flare was seen at 24 h. This peak response did not differ significantly between three and two prior KLH immunizations, which may be explained by the small sample size of the two-times immunization group (n = 5) and should therefore be interpretated with caution (Figure 3). Altogether, these results show a KLH immunization dose-dependency of the cutaneous perfusion response at 48 h, increasing with the number of prior systemic immunizations.

### Estimates of variance analysis

A mixed effects model estimation of the variances was performed to determine the practical implications for the setup of a novel study. With the estimated variances, we determined the impact of the number of KLH immunizations on the number of participants required to detect a significant effect for each of the included systemic and local endpoints of the KLH challenge model (Tables 2,3). In a parallel study design with a power of 0.80 and an alpha of 0.05, an increase in the number of immunizations did not increase the power to detect an immunomodulatory effect on the humoral response, as illustrated by increasing numbers of participants required to detect an effect (Table 2). However, the statistical power of the local endpoints did increase with increasing numbers of immunizations (Table 3). For example, assuming an 80% suppression of both the humoral and systemic responses by a novel compound, three KLH immunizations would require four participants per treatment arm to detect a significant difference in IgM levels and seven participants for flare measured at 24 h, under a parallel study design. A study design incorporating three immunizations and skin imaging at 24 h results in the most robust statistical power.

**TABLE 2:** Sample size calculation–Antibodies.

Inhibition of effect size	IgM		IgG	
	One immunization (N per group)	Three immunizations (N per group)	One immunization (N per group)	Three immunizations (N per group)
10%	386	648	760	1712
20%	87	145	170	381
30%	35	57	67	149
40%	18	29	34	74
50%	10	16	19	41
60%	7	10	12	24
70%	5	7	7	15
80%	3	4	5	9
90%	3	3	3	5
Effect size	301.63	521.13	1,308.24	4,541.54
LOG SD (CV) of the difference	0.520 (CV = 56%)	0.674 (CV = 76%)	0.730 (CV = 84%)	1.096 (CV = 152%)

The effect size was used to calculate the number of participants required to be able to detect a statistically significant effect between two groups in a parallel study design with an alpha of 0.05 and a power of 0.80. The numbers of required participants are presented for an expected inhibition of effect sizes of 10%–90% and compared for IgM and IgG levels after one and three immunizations. The effect size and logarithmic SD used to calculate the number of participants are also shown. Abbreviations: CV, coefficient of variation; SD, standard deviation.

**TABLE 3:** Sample size calculation–Imaging-based endpoints.

Inhibition of effect size	Time after i.d. KLH	Perfusion		Flare		Erythema	
		One immunization	Three immunizations	One immunization	Three immunizations	One immunization	Three immunizations
		(N per group)	(N per group)	(N per group)	(N per group)	(N per group)	(N per group)
10%	24 h		418		334		113
	48 h	6,433	488	4,974	1,416	2,991	3,348
20%	24 h		106		85		268
	48 h	1,609	123	1,244	355	749	838
30%	24 h		48		38		121
	48 h	716	56	554	159	334	373
40%	24 h		28		22		69
	48 h	405	32	312	90	195	211
50%	24 h		18		15		44
	48 h	260	21	200	58	125	135
60%	24 h		13		11		31
	48 h	181	15	140	41	87	94
70%	24 h		10		8		24
	48 h	133	11	103	30	64	70
80%	24 h		8		7		19
	48 h	102	9	79	24	49	54
90%	24 h		7		6		15
	48 h	81	8	63	19	39	43
Effect size	24 h		51.81		39.69		2.33
	48 h	10.78	32.90	6.89	18.08	1.39	0.99
Common SD (AU)	24 h		21		18.3		1.94
	48 h	21.9	18.3	12.3	17.2	1.93	1.46

The effect size was used to calculate the number of participants required to be able to detect a statistically significant effect between two groups in a parallel study design with an alpha of 0.05 and a power of 0.80. The numbers of required participants are presented for an expected inhibition of effect sizes of 10–90% at 24h and 48h after i.d. KLH and compared for cutaneous perfusion, flare, and erythema after one and three immunizations. For one immunization, only the numbers of required participants at 48h are presented. The effect size and common SD used to calculate the number of participants are also shown. Abbreviations: AU, arbitrary units; i.d., intradermal; KLH, keyhole limpet hemocyanin; SD, standard deviation.

### Correlation between systemic and local outcome parameters

**FIGURE 4:**
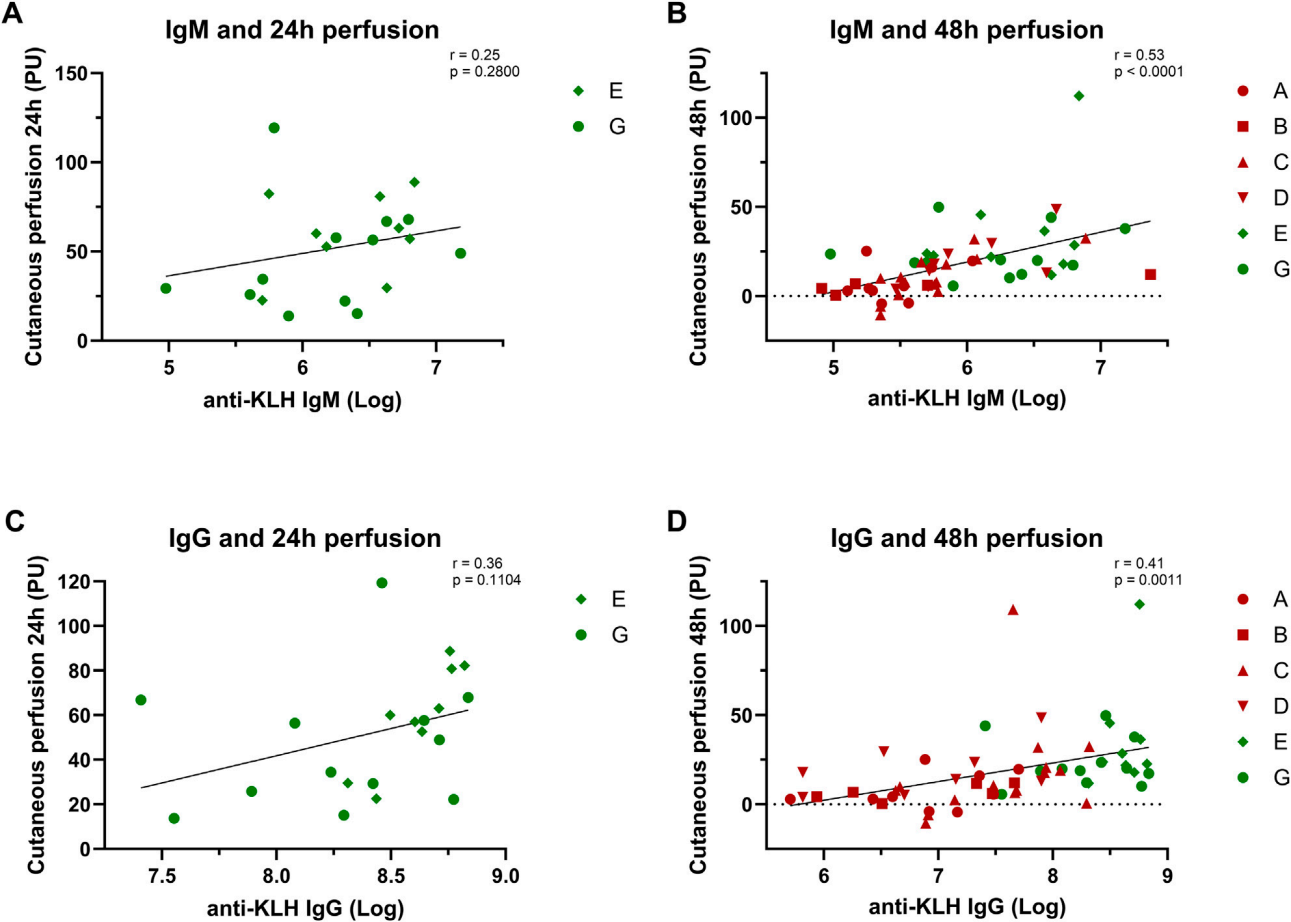
Correlation analyses: Systemic and local response. The correlation between the systemic antibody response and local cutaneous perfusion response is depicted: **(A)** and **(B)** show the correlation between the IgM levels and the perfusion at 24 h and 48 h; **(C)** and **(D)** show the correlation between IgG levels and perfusion at 24 h and 48 h. Studies depicted in dark-red represent studies in which participants were immunized once with KLH. Studies depicted in green represent studies in which participants were immunized three times. Log-transformed values were used for the antibody values to calculate correlations. Pearsons correlation and statistical significance are shown in the corresponding graphs. Abbreviations: h, hours; KLH, keyhole limpet hemocyanin; PU, perfusion units.

We found moderate but significant correlations between the systemic and local immune responses. An increase in antibody levels correlated with an increase in KLH-induced perfusion and flare at 48 h (Figure 4; flare not shown). No significant correlation was found between the antibody response and erythema.

### Covariate analyses: age, sex, and body mass index

**FIGURE 5:**
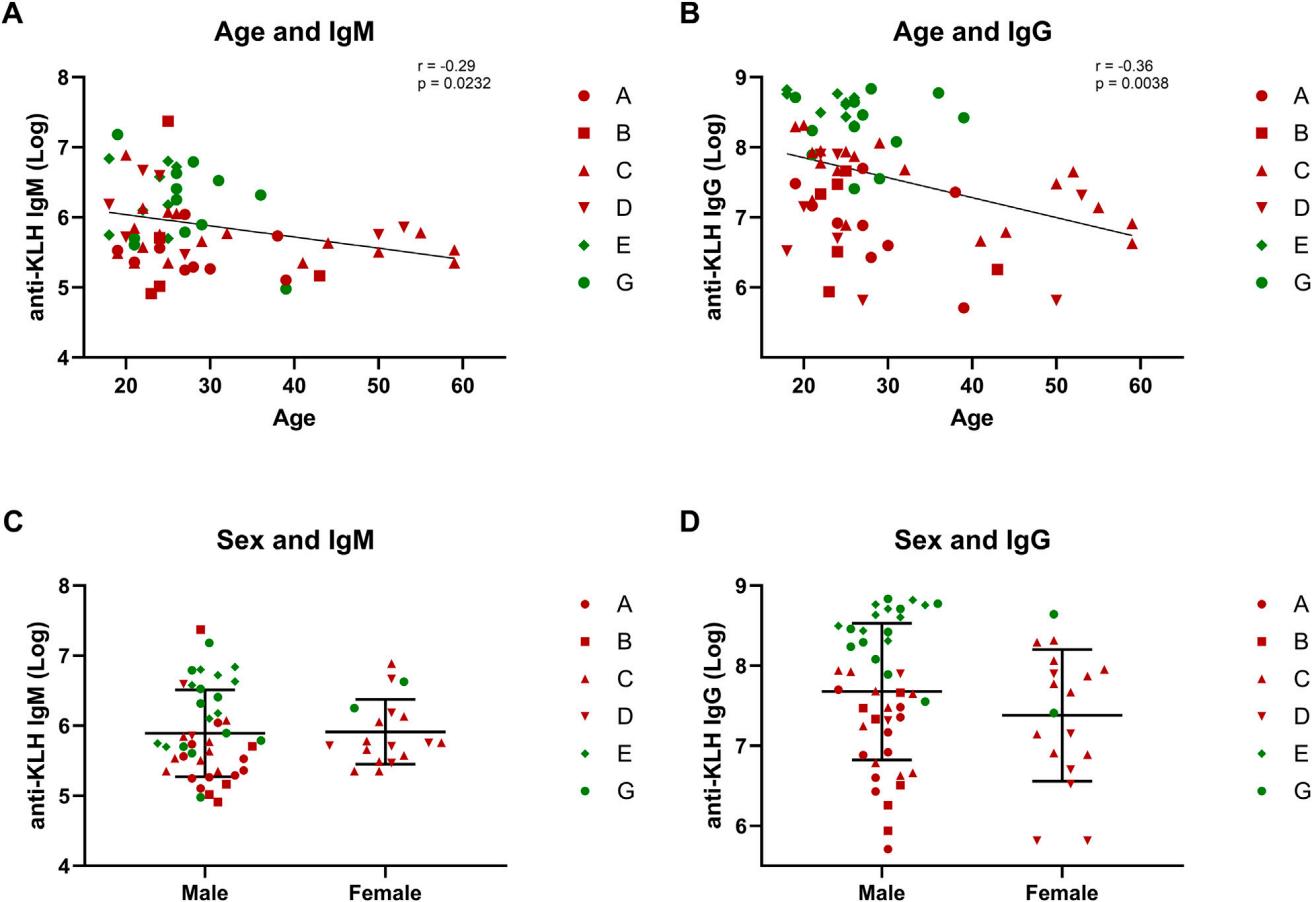
Covariates: Age and sex. The correlations between covariates age and sex and the humoral response are depicted: **(A,B)** show the correlation between age and antibodies IgM and IgG; **(C,D)** form a graphical presentation of the antibody response in males and females. Studies depicted in dark-red represent studies in which participants were immunized once with KLH. Studies depicted in green represent studies in which participants were immunized three times. Log-transformed values were used for the antibody values to calculate correlations. Pearsons correlation and statistical significance are shown in the corresponding graphs. The graphs comparing males and females include the mean ± standard deviation **(C,D)**. Abbreviations: KLH, keyhole limpet hemocyanin.

Of the covariates age, sex, and BMI, both age and BMI were found to be correlated with KLH challenge endpoints. For age, strongly significant correlations were found with IgM (*r* = −0.29, *p* = 0.0232) and IgG (*r =* −0.36, *p* = 0.0038), while for BMI a correlation was found with the local endpoint erythema at 48 h (*r* = 0.25, *p* = 0.0426). No other correlations were found. Sex did not affect any of the systemic or local endpoints, although it should be stressed that the data set was strongly skewed towards males (Figure 5).

## Discussion

The current analysis investigated the KLH neoantigen challenge, in which participants are first immunized with KLH systemically and subsequently administered KLH intradermally to elicit a local recall response. The aim of this analysis based on pooled data from different studies was to evaluate the impact of the number of KLH immunizations on the effect size of the dermal KLH response, quantified by objective non-invasive imaging techniques. The skin response was compared to the systemic response to investigate potential correlations. Furthermore, correlations between age, sex, and BMI and the systemic and local response were investigated to identify covariates impacting the antigen-dependent immune response. The dermal recall response to KLH increased with multiple KLH immunizations. The dose-response relationship between KLH and its outcome parameters was not only seen in the perfusion-directed local recall response, but also in the systemic response. Repeated immunization with KLH resulted in higher KLH-specific antibody titers. The KLH antibody titers decreased with age, while the local response appeared unaffected. KLH-driven dermal perfusion and flare responses were significantly higher after three KLH immunizations compared to a single immunization. The immunization-dependent increase of both systemic and local responses was corroborated by moderate but significant correlations between the systemic antibody response and the local perfusion response.

The increase in the systemic humoral response to KLH based on the number of immunizations is in line with previous research showing incremental increases in antibody titers (Spazierer et al., 2009; Belson et al., 2016). Comparing antibody levels between studies is challenging, not only due to the variety in study design but also due to differences in methodologies. Because KLH is a neoantigen that is infrequently used in clinical practice, no standardized reference material is available (Swaminathan et al., 2014; Saghari et al., 2022). Consequently, we expressed antibody levels as a ratio over baseline and corrected for the impact of the buffer solution on this ratio. This allowed for comparison of the antibody levels of the different studies in this pooled analysis, showing multiple administrations of KLH to lead to higher antibody levels.

The local recall response to KLH was found to be dose-dependent. The findings reported in the current analysis show the KLH-driven skin perfusion response to increase significantly with three prior KLH immunizations as compared to a single immunization. The impact of number of KLH immunizations on the skin response magnitude, based on objective and highly sensitive endpoints, has not been reported comprehensively before (Saghari et al., 2022). Although results of different studies indicate a KLH dose-response relationship based on single immunization regimens, these results are hampered by heterogeneity in methodology and study design. To overcome this issue, the current analysis included seven standardized trials. It should be noted that the sample size at 24 h was limited to five participants for the two-times immunization group. However, when comparing the one- and three-times immunization group at 48 h, there is a convincing impact of the number of KLH immunizations on the KLH skin response.

Following the findings of increased effect size with the number of immunizations, a mixed effects model estimation of the variances was performed to evaluate the impact of repeat immunization on the power of the model to detect modulation of the KLH-driven immune response by compounds in future trials. This analysis showed that the humoral response does not benefit from increasing the effect size by repeated immunization, due to a disproportionate increase in inter-participant variance. However, for the local endpoints, multiple immunizations substantially decrease the number of participants required to detect an immunomodulatory effect of an investigational compound on the KLH response: the evoked skin response increases more than the variance of the response between participants. This is also illustrated by the comparison of the dermal KLH response between 24 h and 48 h: the lowest number of required participants is observed for a scenario with multiple immunizations and a readout at the 24 h timepoint, when the KLH response is largest.

Age significantly affected the systemic KLH-induced immune response, whereas the effect of sex was inconclusive. The age-related decline in KLH-driven antibody responses is in line with immunosenescence, in which immune function gradually declines with age. For the adaptive immune system, this decline primarily originates in thymic involution and shifts in T cell populations (Lange et al., 2003; Lord, 2013; Giefing-Kröll et al., 2015; Liu et al., 2023). Therefore, age was also expected to affect the T-cell-mediated skin response. However, although age correlated with the systemic response, and the systemic and local response were correlated, no correlation was observed between the local response and age. All included studies incorporated age restrictions, the majority limiting participation to individuals under 45 years since in general it is known that the immune response declines with age. Consequently, no definitive conclusions can be drawn regarding the effect of age on the local response. Similarly, the absence of a correlation of sex with systemic and local KLH-driven responses, despite known effects of sex on vaccination responses, was attributed to insufficient variation in the study population (Giefing-Kröll et al., 2015). A larger study population including a wider variation in age and sex will allow for a more comprehensive analysis of the effect of age on the local response. Additionally, this wider variation in the included population is needed to definitively rule out an effect of sex on the systemic and local KLH-driven responses (Vine et al., 2000; İlgen et al., 2022). Until such findings are reported, the current analysis does not provide decisive grounds to distinguish between males and females when performing a KLH challenge. In contrast, the robust association between age and the systemic response supports including age as a key factor in study design and analysis.

The outcomes of this analysis can support the design of future KLH-based studies evaluating the immunomodulatory effect of a novel compound. Increasing the number of immunizations increased the power to detect a statistically significant effect in the planimetric skin response, but decreased the power to detect an effect on the humoral response. Therefore, the focus on humoral or skin responses will determine whether single or multiple immunizations provide the favorable study design.

This pooled analysis was limited to non-invasive skin measurements of the local KLH response. Recently, we reported invasive skin measurements such as flow cytometry on suction blister fluid and immunohistochemistry of skin-punch biopsies to show even more robust KLH-driven responses than imaging-based endpoints (Ronner et al., 2025). Future studies may benefit from including invasive measurements for a more comprehensive understanding of the effects of prior KLH immunizations on the local recall response. Additionally, while we demonstrate three KLH immunizations to yield the most robust cutaneous response, it has not yet been investigated whether this response is equally sensitive to pharmacological modulation. Establishing the extent to which this response can be modulated will provide valuable insights into the translational relevance and clinical applicability of the model. Finally, KLH antibody analyses were limited by the inherent lack of standardized reference material. Developing standardized reference material will benefit future analyses and allow for expression of the KLH antibody levels as concentrations, allowing for easy comparisons between studies.

In conclusion, this pooled analysis provides a basis for the design of future clinical studies with KLH challenges. Depending on the exact study objectives and the mechanism of action of the investigational compound, the KLH immunization regimen, endpoints, sample size, study population, and timing of assessments can be selected based on the body of data presented in this manuscript. Taken together, these data provide further insights into the performance of the human KLH challenge, which will facilitate the future use of this increasingly prevalent immune challenge model.

## Data Availability

The datasets presented in this article are not readily available because portions of the dataset are currently confidential and cannot be shared publicly. Requests to access the datasets should be directed to MM, mmoerland@chdr.nl.
