# Supplementary material for: Neoantigen immune responses in healthy volunteers: insights from multiple integrated keyhole limpet hemocyanin challenge studies on repeated immunization and response covariates
Source: Front Pharmacol. 2026 Jan 2;16:1717333. doi: 10.3389/fphar.2025.1717333 (PMC12808451; doi:10.3389/fphar.2025.1717333)
Supplement: Supplementary file 1 [file Table1.docx]

## Supplementary tables

### Table S1. Study characteristics

Abbreviations: F, female; M, male; y, years

| **Study** | **Immunizations**  **(n)** | **Inclusion** | |
| --- | --- | --- | --- |
|  |  | **Sex** | **Age** |
| **A** | 1 | M | 18-45 y |
| **B** | 1 | M | 18-45 y |
| **C** | 1 | M & F | 18-60 y |
| **D** | 1 | M & F | 18-60 y |
| **E** | 3 | M | 18-45 y |
| **F** | 2 | M | 18-55 y |
| **G** | 3 | M & F | 18-45 y |
